# Development of Australian physical activity and screen time guidelines for outside school hours care: an international Delphi study

**DOI:** 10.1186/s12966-020-01061-z

**Published:** 2021-01-06

**Authors:** Rosa Virgara, Anna Phillips, Lucy Lewis, Mandy Richardson, Carol Maher

**Affiliations:** 1 Allied Health and Human Performance, University of South Australia, c/o GPO Box 2471, SA 5001 Adelaide, Australia; 2 Caring Futures Institute, College of Nursing and Health Sciences, Flinders University, Adelaide, Australia; 3 OSHC SA Chairperson, NOSHA SA Branch, Adelaide, Australia

**Keywords:** Physical activity, Screen time, Delphi, Outside school hours care, Guidelines, GRADE

## Abstract

**Background:**

Children’s activity patterns in the periods before and after school make a key contribution to achieving 24-h movement guidelines. There are currently no national-level guidelines informing physical activity and screen time practices in Outside School Hours Care (OSHC) programs anywhere in the world. This study aimed to work with industry, government and academic stakeholders to develop draft physical activity and screen time guidelines for use in Australian OSHC.

**Methods:**

A 4-round online Delphi survey was conducted from May 2019 to January 2020. The Delphi participants included national and international experts and stakeholders from academia, education, government, health and the OSHC sectors. Round 1 consisted of open-ended questions exploring physical activity, screen time and sedentary behaviour in various periods of OSHC (before school, after school and vacation care). In rounds 2 and 3, participants rated the importance of items generated from the first round for inclusion in national guidelines using a Likert scale (1–9). Consensus was defined a priori as ≥80% of respondents rating an item as “critically important” (score 7–9). Between rounds 3 and 4, the guideline development panel used the consensus items, systematic review evidence, and followed the GRADE process, to draft the guidelines. In round 4, participants were invited to provide feedback on the draft guidelines and comment on barriers and enablers to implementation.

**Results:**

Sixty-seven stakeholders agreed to participate, with response rates 61, 81, 54 and 72% for the four rounds respectively. Of the 123 items generated across the three rounds, 48 statements achieved consensus agreement as critically important for inclusion in the guidelines. These included offering a variety of physical activities (free play, playground and equipment) and restriction of screen time. The final round provided feedback on the draft guidelines. The wording of the guidelines was found to be appropriate and preliminary enablers and barriers to implementation were identified.

**Conclusions:**

This world-first expert and stakeholder consultation has underpinned the development of the draft Australian guidelines for physical activity and screen time in OSHC. Ongoing work is needed to further refine the guidelines, determine current rates of compliance with the guidelines and implement the guidelines into practice.

## Introduction

Widely adopted 24-h movement guidelines recommend that children aged 5 to 18 years achieve 60 min of moderate- to- vigorous physical activity (MVPA) a day, limit recreational screen time to no more than 2 h and achieve at least 9–11 h’ sleep for children aged 5–13 years or 8–10 h for children aged 14–17 years [1–4]. Achieving this balance of physical activity (PA) and sedentary behaviours is linked to a multitude of improved health and wellbeing outcomes for children [5, 6].

As few as 1 in 4 children achieve the recommended amount of MVPA [7] and 1 in 3 meet screen time guidelines [8] . The hours out of school hours are a key time of the day for the accumulation of PA and sedentary behaviours [9, 10]. As many as 18% of children in the US [11] and approximately 10% of Australian children [12] attend outside school hours care (OSHC). This rate is even higher in some countries, such as Norway, where 81% of 6-year-olds attend OSHC [13]. A study of Australian children (mean age 8.1 years) found that the after-school period (3 – 6 pm) accounted for 30% of children’s daily MVPA, 25% of their light PA and 80% of their recreational screen time [10]. Therefore it appears that the OSHC setting holds considerable promise for positively impacting the PA and screen time behaviours of vast numbers of children.

In recent years, some interventions in OSHC have been framed around guidelines for PA in this setting [14–16]. A recent scoping review identified that all guidelines for PA and/or screen time in the OSHC setting have been produced for states or provinces or other regional areas of the US and Canada [17]. None have been national in scope, and none have followed rigorous methodologies during development or publication (i.e. they have not followed rigorous established guideline development methodologies, such as Grading of Recommendations, Assessment, Development and Evaluation (GRADE) [18] or Guideline International Network (G-I-N) [19] methodologies, and all guidelines to date have been published in the grey-literature). In addition, the guidelines to date have varied in the behaviours of interest, (MVPA vs moderate PA vs vigorous PA), whether they cover screen time in addition to PA or not, and whether they cover both before and after school periods. Furthermore, the amount of PA recommendations vary in terms of duration and intensity. The variation in these recommendations, and methodological concerns, make the adoption of these guidelines into other world regions questionable; unlike the 24-h movement guidelines which have consistent messaging and are widely adopted across Australia [1], Canada [2], Croatia [3], Finland [20], New Zealand [4] and South Africa [21] .

This study aimed to address these gaps, by extensively consulting stakeholders and following rigorous processes to develop draft guidelines targeting PA and screen time for use in Australian OSHC.

## Method

### Research design

This was a two-part study. Firstly, an international Delphi survey was conducted to obtain consensus opinion from a range of experts and stakeholders regarding children’s PA and screen time behaviours in OSHC to inform the draft guidelines. A Delphi survey was used as it is a well-established methodology using prospective surveys [22]. It allows for stakeholders to provide insight into important elements for inclusion in the guidelines [22]. Unlike other well established guideline development tools, it was anticipated the Delphi survey would identify issues not apparent in the scientific literature and allow stakeholder engagement and thus potentially result in improved uptake and implementation of the final guidelines [22]. Secondly, the GRADE approach was applied to draft the guidelines. GRADE is used for both clinical guideline development and public health guidelines and is considered the gold standard in guideline development, endorsed and used by the World Health Organisation and NHMRC Australia [23]. A guideline development panel, consisting of the authorship team and four international experts in the field of PA, screen time and implementation research; followed the GRADE approach to draft the guidelines.

The research complies with the recommendations from the Conducting and Reporting of Delphi Studies (CREDES) [24]. Ethical approval was provided by the University of South Australia Human Research Ethics Committee (protocol no. 201786).

### Participant eligibility and panel recruitment

Participants from a range of stakeholder groups involved in the OSHC setting and/or children’s PA and screen time were sought for this Delphi survey. These included national and international researchers (identified as senior authors or renowned researchers in the field of PA and/or screen time), OSHC educators (identified as a Director of an OSHC or senior role in the service), school educators (e.g. school Principals), health professionals (e.g. senior Paediatrician’s within a tertiary health service), government personnel (staff working in a department directly linked to PA) and parents of children attending OSHC services, with an emphasis on geographical diversity so that government and OSHC personnel from all Australian states were included. Full inclusion criteria are provided in Supplementary File 1. Hsu and Sanford [25] recommend that “… if various reference groups are involved in a Delphi study, more subjects are anticipated to be needed” p.3, rather than the usual 15–20 that are used in Delphi surveys [25]. Thus, to ensure representativeness of all stakeholder groups, at least 50 participants were required.

Potential participants were contacted by email outlining the study information (aims and rationale, the time commitment required and a link to the participant information sheet). If the participant opened the first round survey link, they were considered to have shown interest in the study. Completing the survey was considered consent to participate in the study. Participants were encouraged to complete all rounds of the Delphi to minimise attrition and reminded they could withdraw at any stage or continue with subsequent rounds if they had not completed the preceding round.

### Delphi surveys

A maximum of four rounds were planned. Surveys were conducted using LimeSurvey™ software (www.limesurvey.org) [26], with each survey round piloted with people who were not participants in the Delphi proper. Delphi proper participants had 21 days to complete each survey round, with reminder emails sent at days 14, 20 and 21.

### Pilot testing

Round 1 was piloted by four people (2 academics, 1 government personnel and 1 school educator) to ensure appropriateness of the questionnaire items, wording, functionality, and identify any issues or gaps. No changes in wording were made to the first round questionnaire.

### Delphi proper – round 1

**Fig. 1:**
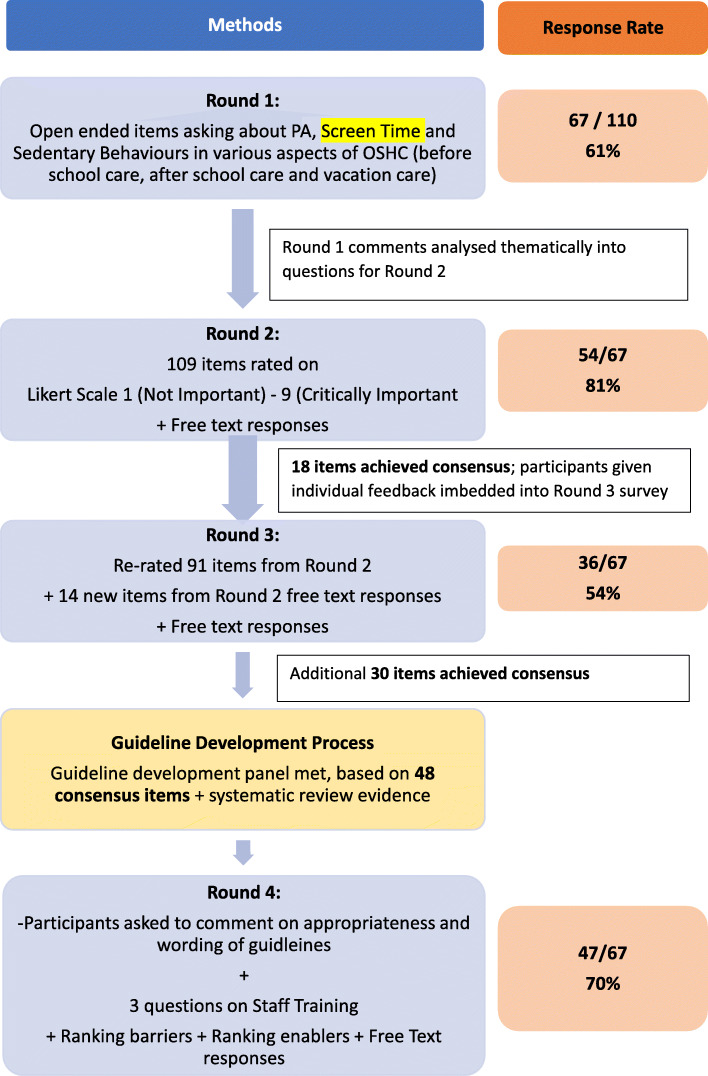
Delphi process in entirety

Round 1 commenced in June 2019. To minimise the potential for personal bias from the research team guiding results [27], the first round focussed on 11 open-ended questions. Participants were provided with an explanation of the survey, and a request for demographic information (including confirmation of email address, location, highest level of education attained, stakeholder group, job title, employer organization, duration of employment, duration of use of OSHC and aspect of OSHC involved in). In addition, they were provided with definitions of OSHC, PA, screen time, before school care, after school care and vacation care. Questions exploring the perceived importance of children’s PA, recreational screen time and sedentary behaviour, across the three OSHC time periods (before school, after school and vacation care) were asked. Round 1 responses were categorised into themes by the lead author (RV) and cross-checked by the research team (AP, LL, CM and MR). A summary of the entire Delphi process is provided in Fig. 1.

### Round 2

Round 2 was developed based on themes from the Round 1 analysis. It comprised of 109 items, relating to PA (type of play e.g. free play/indoor/outdoor; activity intensity e.g. light, moderate, vigorous; session focus e.g. prepare for school day/relax; 24-h movement guidelines), screen time (rules around usage; behaviour management; consistency with 24-h movement guidelines; children and families’ expectations) and sedentary behaviour (type of activities offered e.g. cognitive/social/developmental; 24-h movement guidelines), examined across the three OSHC periods (before school, after school and vacation care). Participants received feedback from the first round consisting of demographic data and tables summarising response rate, key themes and frequency. Participants were invited to rate the importance of each item on a 9-point Likert scale, with 1–3 considered not important, 4–6 important but not critical, and 7–9 critically important. This scoring is consistent with the 9-point GRADE methodology [22]. Examples of items participants were asked to rate were “*How important is it during a before school care session to encourage children to engage in outdoor active play” and “How important is it during an after school care session to provide tight limits for recreational screen time use?”* Consensus was defined a priori as ≥80% participant agreement, meaning ≥80% of respondents had to rate the statement as being ‘critically important’ (i.e. ≥ 80% or more of scores were between 7 and 9) for inclusion in the draft guidelines. Participants were also invited to provide any further comments after rating the item in each of the categories.

### Round 3

**Fig. 2:**
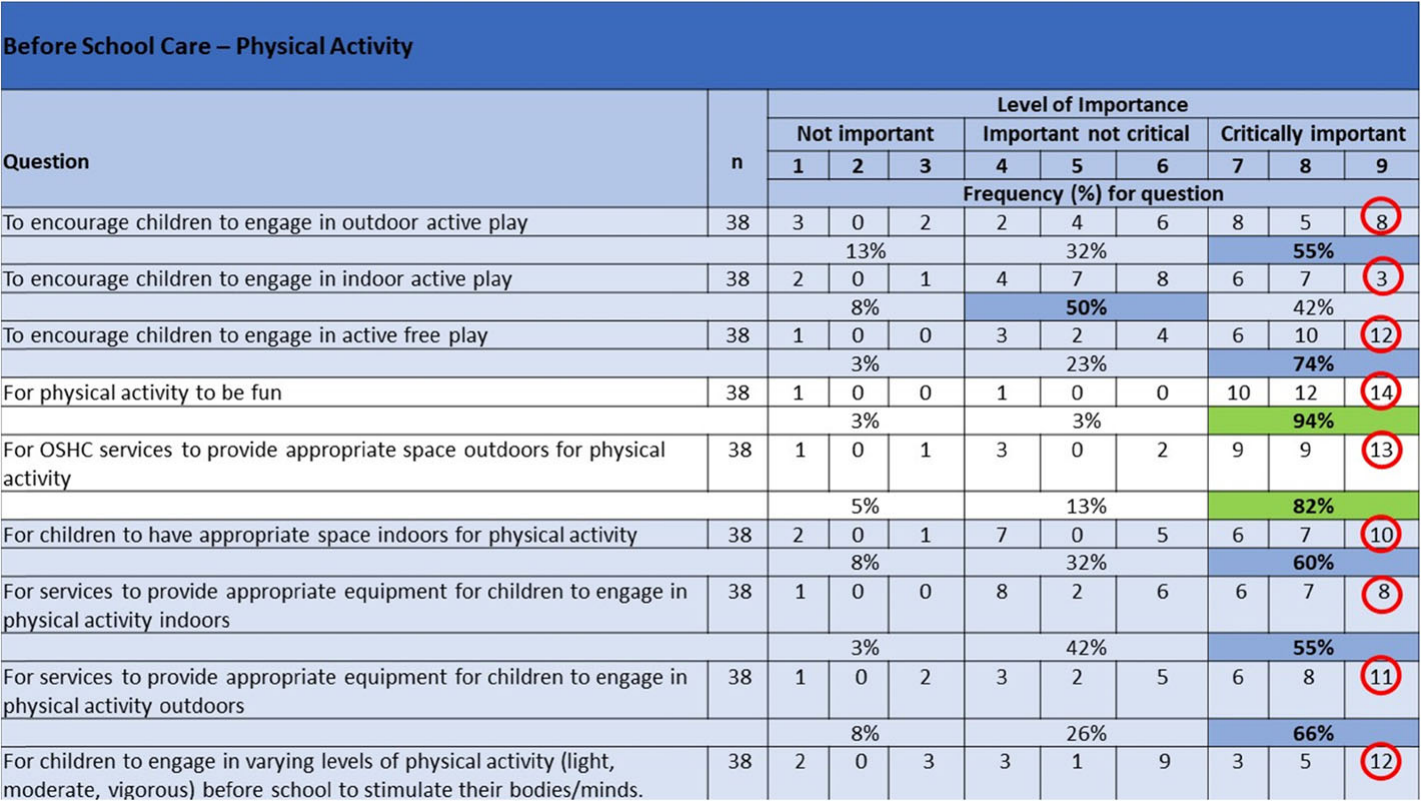
Example participant result from round 2 embedded in round 3 Survey

Round 3 was developed from Round 2 analysis and administered in September 2019. Participants were invited to re-rate items which had not achieved consensus in Round 2; and invited to rate new items that were generated from free-text responses in the previous round. Participants were provided with a reminder of definitions, along with individual and group scores from Round 2 embedded into the survey to reduce participant burden and assist with re-rating (Fig. 2).

### Guideline development process

On completion of Round 3, and as part of the GRADE process, the Guideline Development Panel met and drafted the guidelines using the items that achieved consensus from rounds 2 and 3 of the Delphi survey. These consensus items were used with the results from the scoping review of PA and screen time guidelines in OSHC [17] and the results of the systematic review of interventions to increase PA in OSHC [28]. These draft recommendations consisted of two components. The first section contained a “snapshot” guide which provided a quick reference for OSHC personnel to identify what time targets they should aim for in each session of OSHC. The information obtained from the reviews was used to inform the time targets. The second section was the elaboration document. This included a preamble providing the health effects associated with PA and screen time, a reference to the 24-h movement guidelines and how the OSHC environment can help children achieve the 24-h movement guidelines. The elaboration document made use of the items that met consensus in the Delphi to inform the content. These consensus items were incorporated into sections under the headings of PA recommendations, screen time recommendations and recommendations for educators.

### Round 4

Round 4 commenced in December 2019 and closed in January 2020 and consisted of 13 questions. This included seven open-ended questions seeking comments on the wording of the draft guideline document, the durations recommended for PA and screen time and any additional comments. In addition, four multiple-choice questions asked about preferences for staff training on the guidelines (delivery mode, session length, educational materials), and two questions asking participants to rank the top three barriers and enablers to implementing such guidelines into practice. A final opportunity for any further comments was also provided.

### Data analysis and management

Study uptake was calculated as the percentage of participants who opened the Round 1 Delphi survey from all potential participants contacted. Study retention rate was described as the percentage of participants completing the surveys and was calculated separately for each Delphi round. Participant characteristics were reported descriptively. For Likert scale items, all responses were used to calculate the mean, standard deviation (SD), median and mean absolute deviation from the median (MADM) (describes the variation in the median values) [29]. For consensus to be achieved, ≥ 80% of respondents must have rated the statement as being ‘critically important’ (i.e. Likert score between 7 and 9).

## Results

### Delphi proper

A total of 110 potential participants were identified and invited to be involved in the Delphi survey. Of those, *n* = 67 provided informed consent (OSHC educators (*n* = 22), Parents (*n* = 14), Researchers/Academics (*n* = 13), Government/NGO personnel (*n* = 8), Other (*n* = 5), unanswered (*n* = 5)), leading to an uptake rate of 61%. Of those who didn’t participate, *n* = 6 declined participation and *n* = 37 did not respond. The response rate for each round ranged from 54 to 81% (Fig. 1). All Australian states and territories were represented. The majority of participants were from Australia 87% (*n* = 54) and 13% (*n* = 8) of participants were international. Most participants had a Bachelor degree or higher (71%, *n* = 44), with a further 26% holding a graduate or trade certificate (*n* = 16).

### Round 1

Participants were asked to provide comments about PA, screen time and sedentary behaviour across the three OSHC periods (before school care, after school care, and vacation care). Common themes that emerged were related to 24-h movement guidelines (*n* = 10), activities (*n* = 6), children and families (*n* = 6), rules (*n* = 3), staff (*n* = 3), type of play (*n* = 2), purpose of session (*n* = 2), and behaviour and training (*n* = 1) respectively. Themes that were common to all aspects across all OSHC time periods, were that recommendations should help children achieve the 24-h movement guidelines, and finally that staff, children and families should be involved in the implementation of such recommendations. Themes that were related to PA included type of activity (i.e. play-based PA, enjoyment of PA, and allowing for varying intensities of PA) and guidelines (24-h movement guidelines considered). The most common screen time theme regarded rules (i.e. limitations on quantity and content of screen time). Sedentary behaviour themes were also centred around 24-h movement guidelines and activity (i.e. they should allow for self-regulation, and play-based activities). Staff role modelling to encourage positive PA and screen time behaviours in the OSHC setting was prominent. Examples of participant statements are provided in Table 1:

**Table 1:** Example Delphi participant open responses from Delphi rounds 1 and 2

Delphi Round	Category	Delphi Participant Comment
1	PA – before school care	*“It’s important for kids to wake up their mind and body before they start the day at school. Any form of physical movement stimulates the body and mind, making the kids more receptive to learning. And it’s also fun.”*
	Screen time – after school care	*“As little as possible. If screen time is used this should be active screen time (*e.g. *active video gaming or geocaching).”*
	Sedentary Behaviour – after school care	*“Activities such as reading, board games construction/building activities should still be available for use. While they may still be of a sedentary nature, they provide more opportunity for both social interaction and learning compared to recreational screen time alone. However, still monitor the more sedentary activities and encourage children to mix it up with outdoor active play.”*
2	Screen time – after school care	“*If screens are offered children will watch. Limit the offering and limit the watching. Don’t want to demonise it, the better approach is to offer more appealing alternatives that have a more active component.”*
	Screen time – vacation care	*“As stated we do not use screens as much as possible and have seen a big change in behaviour and attitudes from children”.*

### Round 2

Round 2 of the Delphi survey consisted of 109 statements, consensus agreement was reached for 18 items, 13 relating to PA, two to screen time and four to “other” (scoring between 7 and 9 “critically important”) to include in the guidelines (Table 2). Examples of the 13 PA items that reached immediate consensus as being critically important to include were “Physical activity to be fun” and “To provide opportunities for unstructured/free play to encourage physical activity” which achieved agreement of 94 and 97% respectively. An example of the screen time items that reached consensus was “That staff and older children model appropriate screen time use”. No sedentary behaviour items achieved consensus in the first round.

**Table 2:** Items achieving consensus agreement from Delphi Round 2

Statement	Responses (n) All round 2 ***n*** = 54	% agreement (≥ 7 on 9 point Likert scale)	Mean Likert score (SD)	Median (MADM)
***Before School Care***				
***Physical Activity***				
For physical activity to be fun	43	95%	7.9 (1.5)	8 (0.9)
For OSHC services to provide appropriate space outdoors for PA	43	81%	7.3 (1.9)	8 (1.4)
***Screen Time***				
That staff and older children in OSHC services role model appropriate screen time use e.g. only access personal devices at beginning and end of sessions	43	80%	7.5 (2.0)	8 (1.6)
That recreational screen time should be limited	43	81%	7.2 (2.2)	7 (1.6)
***After School Care***				
***Physical Activity***				
To provide an opportunity for free play/unstructured physical activity	39	95%	8.2 (1.1)	9 (0.9)
To provide fun physical activity	39	92%	8.2 (1.2)	9 (1.0)
To provide opportunity for outdoor play and outdoor equipment to encourage physical activity	39	92%	8 (1.1)	8 (0.9)
To provide physical activity opportunities for children of different ages	39	89%	8.1 (1.2)	9 (0.9)
To provide physical activity opportunities for children of different abilities	39	95%	8.3 (1.1)	9 (0.9)
To provide opportunities for varying intensities of physical activity (light, moderate, moderate-vigorous, vigorous	39	84%	7.5 (1.7)	8 (1.2)
***Screen Time***				
That physical activity is the preference to recreational screen time	39	82%	7.6 (1.6)	8 (1.3)
***Vacation Care***				
***Physical Activity***				
To provide a diverse range of physical activity throughout the day	38	89%	7.8 (1.4)	8 (1.0)
To provide opportunities for outdoor physical activity	38	97%	8.2 (1.1)	9 (0.9)
To provide opportunities for unstructured/free play to encourage physical activity	38	97%	8.3 (1.0)	9 (0.9)
To provide physical activity that is fun	38	97%	8.3 (0.9)	9 (0.8)
***Other***				
That OSHC educators are continually supported, upskilled and motivated to help children achieve healthy activity behaviours	36	86%	7.5 (1.2)	7 (1.0)
To provide a wide range of activities throughout the day in OSHC services with recognition of the 24 h movement guidelines (i.e. balanced between physical activity, sedentary behaviour and screen time	36	83%	7.6 (1.4)	8 (1.1)
To use a guideline or policy to better guide physical activity, recreational screen time and sedentary behaviours in OSHC	36	81%	7.6 (1.3)	8 (1.1)

*MADM* Mean absolute deviation from median, *SD* Standard deviation

Free text responses were provided by 18 participants. Themes were similar to Round 1, including activities, guidelines and rules. Free text responses for PA included providing appropriate provocations to encourage PA. Themes about rules were again prominent in responses to recreational screen time, and included free text responses about limiting screen time, particularly in after school care to counter the amount of screen time children have at home and during a school day. Some example comments are also provided in Table 1.

Themes in sedentary behaviour were around the benefits of sedentary activities (cognitive, social) and encouraging children to self-regulate.

### Round 3

Round 3 consisted of 105 items for participants to rate (91 items that did not achieve consensus agreement and a further 14 new items developed from free-text responses provided by participants in Round 2). A further 30 items reached consensus as being “critically important” to include in the guidelines at the end of Round 3 (Table 3). Twelve items were relating to PA, six to screen time, ten to sedentary behaviour and a further two in the “other” category. Key PA items across all time periods of OSHC included that PA should be fun, incorporate free/unstructured play, indoor and outdoor play, opportunities for varying ages and abilities and for OSHC educators to consider the 24-h movement guidelines when scheduling PA at OSHC. Key screen time statements included limiting screen time, engagement with families and children about screen time practices in OSHC and ensuring vacation care does not exceed 24-h guideline limits. Sedentary behaviour items across OSHC time periods were strongly focussed around balancing between PA and quiet sedentary behaviour activities, with importance on encouraging children to self-regulate and move between activities. “Other” items that reach consensus focused on education and support provided to OSHC staff such as training and use of a policy or guideline document specifically for OSHC, incorporating elements of the Health Physical Education curriculum and integrating the 24-h movement guidelines when planning OSHC sessions.

**Table 3:** Items achieving consensus agreement from Delphi Round 3

Statement	Responses (n) Participated Round 3 ***n*** = 36	% Agreement	Mean (SD)	Median (MADM)
***Before School Care***				
***Physical Activity***				
To encourage children to engage in active free play	34	80%	7.5 (1.5)	8 (1.2)
To provide quality environments that enable free active play^#^	34	85%	7.7 (1.2)	8 (1.0)
To provide indoor or outdoor provocations to encourage active play^#^	34	80%	7.3 (1.4)	7 (1.0)
To encourage active play to provide social benefits^#^	34	82%	7.5 (1.4)	8 (1.1)
To provide activities which encourage children to be active^#^	34	91%	7.9 (1.2)	8 (1.0)
***Screen Time***				
That active video gaming is allowed^a^	33	85%	2.2 (1.5)	1 (1.3)
***Sedentary Behaviour***				
To provide opportunities for free play	31	90%	7.8 (1.7)	8 (1.1)
To provide children the option to choose their activities/self-regulate	31	81%	7.2 (1.8)	7 (1.3)
***After School Care***				
***Physical Activity***				
For staff to have ongoing training support on ways to embed physical activity into policy	30	87%	7.7 (1.4)	8 (1.2)
For staff to have ongoing training about the benefits of physical activity	30	87%	7.8 (1.2)	8 (1.0)
***Screen Time***				
That there is engagement between staff and children and their families about the effects of screen time	30	80%	7.1 (2.1)	7 (1.4)
That the screen time recommendations in the 24 h movement guidelines are considered when timetabling activities.	30	80%	7.3 (1.9)	7.5 (1.4)
***Sedentary Behaviour***				
That sedentary activities are balanced with physical activity during after school care	28	89%	7.6 (1.1)	7 (1.0)
That staff engage with children to help them move between activities (e.g. if spent a long time in sedentary activities move to a physical activity, or if need to calm down participate in a sedentary task)	28	86%	7.4 (1.3)	7 (1.0)
That children are encouraged to choose/self-regulate their sedentary activities	28	82%	7.4 (1.5)	7 (1.2)
To provide balanced periods between active and sedentary play	28	82%	7.4 (1.3)	7 (1.1)
***Vacation Care***				
***Physical Activity***				
To provide opportunities for indoor physical activity	27	81%	7.3 (1.6)	7 (1.1)
To balance the program between physical activities and sedentary activities	27	89%	7.5 (1.4)	7 (1.1)
To allow children the opportunity to choose their activity	27	81%	7.6 (1.7)	8 (1.3)
To consider the 24 h movement guidelines when scheduling the day	27	85%	7.4 (1.7)	8 (1.3)
To educate staff and children re: benefits of the 24 h movement guidelines and importance of achieving them	27	85%	7.4 (1.8)	8 (1.3)
***Screen Time***				
To involve children and families in decision making around recreational screen time practices	27	81%	7.3 (2.2)	8 (1.6)
To provide education to children and families about the effects of excessive recreational screen time	27	89%	7.7 (1.8)	8 (1.2)
To ensure vacation care does not exceed the recreational screen time guidelines of the 24 h movement guidelines	27	89%	7.7 (1.8)	8 (1.3)
***Sedentary Behaviour***				
That sedentary activities are balanced with physical activity	27	85%	7.6 (1.2)	8 (1.0)
That sedentary activities are available to allow “rest and recharge”/ “wind-down” time	27	81%	7.2 (1.5)	7 (0.8)
That staff carefully plan the session to consider the needs of the children	27	96%	7.8 (1.6)	8 (1.1)
That ongoing staff training and education is provided to ensure a balanced session with a student centred approach	27	93%	7.7 (1.3)	8 (1.1)
***Other***				
That OSHC educators are provided ongoing training and education to develop the Health and Physical Education curriculum in OSHC	27	85%	7.2 (1.7)	7 (1.1)
To provide children and families with information of the health benefits of following the 24 h movement guidelines	27	89%	7.4 (1.7)	7 (1.1)

*MADM* Mean absolute deviation from median, *SD* Standard deviation # New Question to Round 3, ^a^ Rated NOT IMPORTANT, *SD* Standard deviation, *MADM* Mean absolute deviation from mean

### Guideline development process

**Fig. 3:**
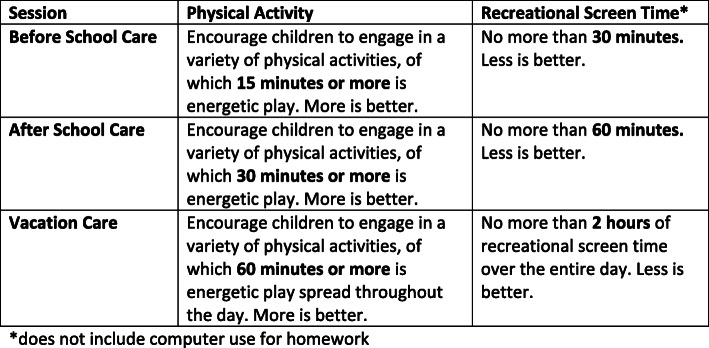
First Iteration of Snapshot Guidelines

Following the conclusion of Round 3, the guideline development panel met on four occasions in November and December 2019, to draft the guidelines. Care was taken to balance data from the Delphi, including priorities, feasibility for the sector (based on feedback from industry advisor and comments in the Delphi) and including scientific content from the reviews (17, 28). The draft guideline document was created by the guideline development panel, and included a snapshot of the guidelines (Fig. 3) and the expanded guideline document (Supplementary File 2).

### Round 4

Overall the draft Australian PA and screen time guidelines were considered by Delphi participants to provide appropriate durations of time recommended for the activities based on free-text responses (*n* = 25) agreement statements (e.g. “*Agree”, “Appropriate to me”, “like the wording”).* Of those who had concerns about the recommended time targets (*n* = 13), their comments included *“Screen time in the afternoon should be no more than 30 minutes (versus 1 hour)”* and “*The physical activity times stipulated for both before and after school care could be challenging for children who are not there for the full session - some children may only attend after school care for 30 - 60 minutes so by the time they get there have a snack/afternoon tea etc they may not have time to do the suggested physical activity quota.”* Some participants did not provide any free text responses at all (*n* = 9).

The top three ranked barriers to implementing the guidelines were 1) educator beliefs, 2) workplace culture and 3) children’s beliefs. The top 3 enablers to implementation were 1) appropriate staff training, 2) ensuring educator understanding and 3) workplace policy. Online education/training sessions with electronic resources were the preferred methods of resource delivery, with a duration of 60 min.

**Fig. 4:**
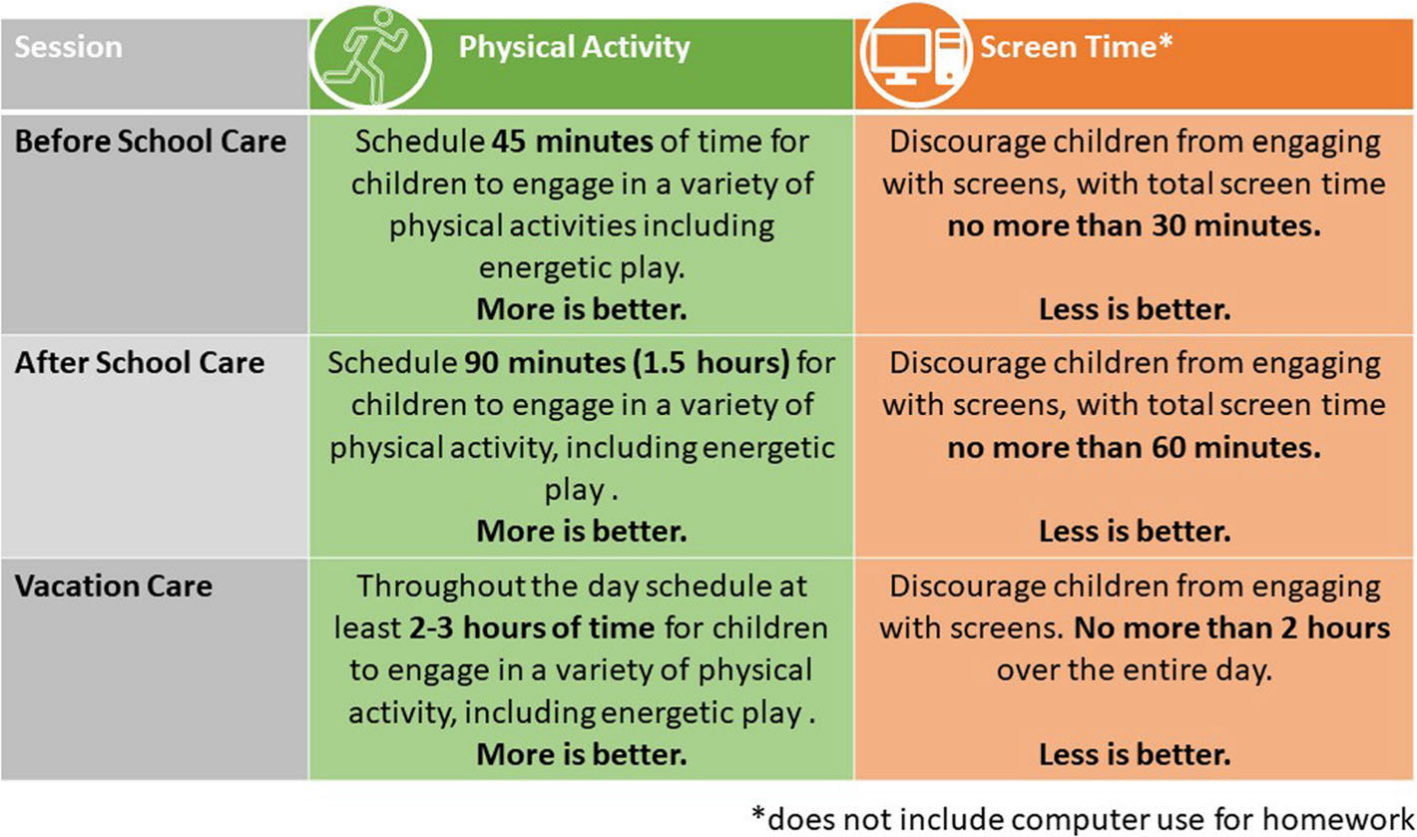
Final Draft Physical Activity and Screen Time Guidelines for OSHC

Following Round 4, the guidelines were refined based on feedback. In particular, to address the concern that it would be impossible for OSHC directors to ensure all children achieved the PA components of the guidelines, the guidelines were reframed so that they were aimed at the OSHC service level rather than the child level. This meant that, instead of the guidelines stating how much PA each child should receive (which is beyond the OSHC director’s control, due to child-level factors such as when their caregiver delivers them/collects them from OSHC), the refined guidelines stated how much PA opportunity the service should provide. In doing this, a ratio of 3:1 was selected, based on evidence from previous research in the sector [30, 31]. The final iteration of the OSHC PA and screen time guidelines is shown in Fig. 4.

## Discussion

This study used an international consensus Delphi survey to inform the development of PA and screen time guidelines for Australian OSHC services. This was achieved using key concepts identified and valued by Delphi panel members, combined with the GRADE approach incorporating evidence from recent systematic and scoping reviews [17, 28]. A range of experts in the field of PA and screen time were involved in the Delphi surveys, with significant input from stakeholder groups such as OSHC educators and families. The Delphi survey identified several key concepts that were applied in the draft guidelines. The main PA concept across all aspects of OSHC was that it should be play-based and fun, with ample opportunity for free active outdoor play. Screen time, if offered at all, should be limited and balanced with PA, whilst quiet sedentary activities should be available to allow children to self-regulate and move freely between active and quiet play. To bring about these changes, OSHC staff should be supported with training to assist them to facilitate PA effectively. Engagement with children and families to help them understand and accept changes (particularly to screen time) will be needed.

Results highlighted that PA in the OSHC setting should be flexible and play-based, rather than PA being structured and run like a physical education class. OSHC occurs during what is normally children’s discretionary time i.e. time when children are free to engage in their own pursuits [32]. By providing a guideline that supports children’s free play, it enables them to self-regulate, which was an important concept identified in the Delphi process and consistent with the evidence that play-based physical activity is associated with improved social and cognitive development [33–35]. The emphasis on free play in Australian OSHC is consistent with findings from a recent observational study in OSHC in Norway, which reported that over half of children’s MVPA was accumulated during outdoor free play [36].

The Delphi survey identified that screen time in OSHC needed limits or rules to govern its use in the setting; with attention to the quantity, quality and the content of screen time identified as important factors. The statement that “Screen time should NOT be a part of OSHC” (i.e. that there should be no screen time at OSHC), did not achieve consensus. While it was strongly supported by some (including OSHC directors whose own services had successfully removed screen time) others (including OSHC directors) strongly wanted screen time in moderation. Some participants stated they were concerned about the amount of screen time children engage in during a school day and once they are home, and that quality OSHC programs shouldn’t need to rely on screen time to provide an enjoyable program. If screen time was offered, it could be educational in nature or active gaming e.g. Nintendo Wii or Just Dance programs. Previous literature has highlighted the benefits of active video gaming, demonstrating an improvement in overall PA, mood, self-esteem and other cognitive and academic outcomes (attention, visual-spatial skills, academic performance [37]. By contrast, some Delphi participants stated access to screen time was an incentive for children to attend OSHC, as this wasn’t available to them at home. It is unclear whether such comments referred to screens not being available by family choice, or due to socioeconomic reasons. Previous Australian research has identified high recreational screen time device ownership amongst children from low SES families [38]. However, it also draws attention to the idea that Australian families using OSHC families are essentially consumers, and OSHC is a product. In Australia, OHSC is most commonly used by working families, whom require care of their children outside of school hours; compared to OSHC in other countries, such as the US, in which OSHC is used for academic enrichment and caters more for low SES children [39]. If children and families using OSHC are consumers, then OSHC services directors may feel pressure to provide programming that children want, so that families will continue to use the service. This may be a barrier to reducing the use of screens in OSHC. Given the contention over the no-screen Delphi item, it makes sense that the statement about providing some screen time in OSHC within limits and rules achieved consensus instead.

Another important point from the screen time comments in the Delphi was that the 24-h movement guidelines need to be considered when programming screen time, particularly for vacation care. Vacation care sessions run during school holidays for an entire day. The Delphi achieved consensus that the 24-h guidelines should not be exceeded during these sessions. This statement is consistent with research that has demonstrated when children achieve the recommended balance of PA, recreational screen time and sleep, as per the 24-h movement guidelines, they have improved cardiorespiratory fitness, health related quality of life and reduced BMI [40]. Rather than banning screen time altogether during this session, it should be balanced with PA and cognitive and social sedentary tasks such as puzzles and board games, which have been demonstrated to have associations with higher academic performance such as writing, numeracy, language, reading and spelling [41].

The Delphi was able to identify important factors related to staff. In particular, the statements that pertained to the importance of staff modelling appropriate screen time behaviours, helping children to move between activities, and having a policy or guidelines to help them plan appropriate PA, screen time and sedentary behaviours in OSHC all reached immediate consensus. This too is consistent with previous research which has found that interventions in a US OSHC setting were more effective when staff display and model behaviours to support healthy eating and PA [42].

Finally, the statement on need for OSHC staff to understand the 24-h movement guidelines and the associated health benefits also reached immediate consensus. While this underscores the *importance* of knowledge of the Australian children’s 24-h movement guidelines amongst OSHC staff, we don’t know what the actual *level of awareness is* at present. Certainly, in other sectors, awareness of physical activity guidelines amongst clinicians and practitioners has been shown to be surprisingly low (for example, a 2017 survey of Australian physiotherapists found that only 10% could accurately state the physical activity and sedentary behaviour guidelines [43]). Future research examining awareness of the 24-h guidelines amongst OSHC staff, and potentially addressing deficits, is warranted. The many consensus items from Rounds 1–3 of the Delphi informed the draft Australian PA and screen time guidelines for OSHC. They provided important information and ideas that could not have been identified in the scientific literature [22].

### Strengths and limitations

This study had several strengths. The Delphi component of the study was conducted in a rigorous manner, with high participant response rates and representation from a range of national and international experts, stakeholders and end-users. Four rounds of the Delphi were conducted over 7 months with an average response rate of 67%. This result is consistent with a recent systematic review from Gargon et al. [44] which found Delphi response rates varied from 45 to 100%. In addition to this, this Delphi survey was novel in that a large proportion of the participants were end-users (OSHC educators and parents combined represented 51% of the survey participants). This is a change to traditional Delphi surveys, which typically are for clinical purposes e.g. in palliative care Delphi techniques are used to develop clinical guidelines for treatment recommendations and assessment tools when it is inappropriate to use other typical research methods (i.e. RCTs, observational studies) [24] . This is a novel approach to a Delphi technique and allows for crucial end-user engagement, and is becoming more apparent in the literature [45, 46]. This is consistent with contemporary population health research approaches, which recognise that best practice is to involve patients or the public with research that not only helps to identify appropriate priorities, but is more relevant to user needs [47]. The Delphi also identified barriers and enablers to implementing such a guideline into this setting. These were factors such as educator beliefs and workplace culture. This is an important step for future implementation research, as it has provided some initial data that organisational issues are likely to be a barriers to implementation [48]. Practical information was also obtained about the best way to engage with OSHC educators through training workshops, approximately 60 min in duration with ongoing access to resources and support. This is important, as the turn-over of staff in the OSHC setting is high, and professional development opportunities limited. Thus, any resources that are developed need to be easily accessible, time-efficient and easily understood [49].

The second component of this study was the development of draft Australian PA and screen time guidelines. This followed the GRADE process, which is considered the gold standard in guideline development [23] and allowed for not only the use of evidence from systematic reviews, but also incorporated the consensus items from participants of the Delphi. This approach allowed for further stakeholder input into the guidelines and contributes to the strength of the draft guidelines. This was reflected in the results from Round 4 when the wording and recommendations of the draft Australian PA and screen time guidelines were well received by participants, with few concerns over the time durations recommended and many positive comments on the appropriateness of the wording.

Some limitations to this Delphi survey were that despite the rigour in method used and the best efforts of the research team to engage participants (using an online forum, with several reminder emails and short survey duration), not all responses were complete, meaning data were sometimes missing from some questions. By design, the Delphi panellists were invited to ensure broad representation of all Australian states and stakeholders, with a view to generating results that are generalisable to the national OSHC context. It is possible that results don’t truly represent specialised OSHC settings, for example, OSHC services in remote areas, and OSHC services serving specialised child populations such as single-sex schools, children with disabilities, or services with high numbers of children from different cultural backgrounds.

#### Implications for future research

The findings of this study show draft Australian PA and screen time guidelines developed for use in OSHC are welcomed by the sector and the setting has a role in helping children to achieve the 24-h movement guidelines. The use of these guidelines may provide a benchmark for services to aim for during OSHC sessions and a way in which they can continually improve practice through self-assessment against the guidelines. The draft guidelines also support the Australian National Quality Framework standards that Australian OSHC services need to meet – particularly standard 2.1 “That children’s health and physical activity are supported and promoted” [50]. Future research should build on this initial work, by further refining and assessing the guidelines through end-user validation, followed by widespread implementation and dissemination assessment of these guidelines into daily OSHC practice. Key barriers and enablers were also identified from this study relating to workplace culture and children’s expectations in OSHC, which will be important to consider in future research.

## Conclusion

Extensive engagement, representing a variety of stakeholders, including strong representation from end-users, resulted in a series of consensus statements regarding PA and screen time in OSHC setting, which informed the development of draft Guidelines for PA and screen time guidelines in Australian OSHC. These guidelines will provide a benchmark for OSHC services to provide quality PA and screen time programming. Training and support to assist OSHC staff to facilitate quality PA is needed and should be designed to acknowledge the constraints of OSHC workforce (characterised by high levels of casualisation, and staff turn-over). In addition, involvement of children and parents will be important to aid the acceptability of that changes in PA and screen time practices in OSHC services.

## Supplementary Information

**Additional file 1.**

**Additional file 2.**

**Additional file 3.**

## Data Availability

The datasets used and/or analysed during the current study are available from the corresponding author on reasonable request.

## Abbreviations

MVPA: Moderate – to- vigorous physical activity
PA: Physical activity
OSHC: Outside school hours childcare
