# Supplementary material for: Development of Australian physical activity and screen time guidelines for outside school hours care: an international Delphi study
Source: Int J Behav Nutr Phys Act. 2021 Jan 6;18:3. doi: 10.1186/s12966-020-01061-z (PMC7789289; doi:10.1186/s12966-020-01061-z)
Supplement: Supplementary file 1 — Additional file 1. [file 12966_2020_1061_MOESM1_ESM.docx]

| **Stakeholder** | **Inclusion criteria** | **Recruitment Strategy** | **No. invited** | **No. participated** |
| --- | --- | --- | --- | --- |
| Academics and researchers | Professorial or Associate Professorial level | Researchers with international reputation for expertise in children’s physical activity and screen time **and/or**  Researchers with expertise in children’s physical activity or screen time in the after school care setting **and/or**  Senior authors on key background literature e.g. Australian Physical Activity Guidelines **and/or**  Identified through professional contacts **and/or** based anywhere worldwide | 25 | 13 |
| Parents | Must be a current user of an OSHC service | Identified through professional contacts from OSHC Industry supervisor **and/or**  Identified from Facebook advertisement (see Appendix 1) | 25 | 14 |
| Government sector | continued employment in the area for at least 2 years to ensure adequate understanding/background knowledge | Staff working in a department directly related to children’s health and well-being i.e. Paediatricians in tertiary hospitals, either a health department, department of education, department of sports and recreation or other departments that are related (e.g. commission for children and young people) as identified by their title and office through online searching **and/or**  Identified through professional contacts from supervisory panel e.g. Department of Education colleagues **and/or**  To ensure representation Australia wide, if a state or territory has not been identified, those websites e.g. education.sa.gov.au will be searched to find appropriate personnel in positions related to the project e.g. a project coordinator for children’s health and wellbeing in the department of education | 24 | 8 |
| Education sector | Senior role within the facility e.g. director of service or second in charge | Based anywhere in Australia **and**  Director of an OSHC service for at least 2 years **or** OSHC educator who has worked in an OSHC service for at least 2 years **or** involved in school-based policies e.g. Principal, curriculum authority | 25 | 22 |
| Private sector | Senior role within the facility e.g. director of service or second in charge | Those organisations who run OSHC facilities privately e.g. Camp Australia will have their state delegates approached as identified through website details/contacts. | 11 | 5 |
