# Supplementary material for: Development of Australian physical activity and screen time guidelines for outside school hours care: an international Delphi study
Source: Int J Behav Nutr Phys Act. 2021 Jan 6;18:3. doi: 10.1186/s12966-020-01061-z (PMC7789289; doi:10.1186/s12966-020-01061-z)
Supplement: Supplementary file 2 — Additional file 2. [file 12966_2020_1061_MOESM2_ESM.docx]

**PREAMBLE**

It is important for children to engage in appropriate amounts of physical activity to achieve health benefits and enhanced well-being. Moderate to vigorous physical activity i.e. activity which makes children huff and puff such as running, jumping, playground play, ball games, provides the greatest health related benefits. Recreational screen time such as TV viewing/movies/computer use/video consoles/personal devices, is associated with poorer health outcomes such as increased anxiety, sleep disturbances and reduced cognitive performance.

OSHC services provide a unique opportunity for school aged children to engage in activities that they are unable to during a structured school day. These recommendations and guidelines have been developed as a way to encourage children to be active through free play and engaging in programmed activities can help them achieve optimal health and well-being.

The following guidelines and recommended strategies have been developed using the best available research evidence, expert agreement and stakeholder input to guide children’s physical activity and screen time practices in OHSC services.

**GUIDELINES IN A SNAPSHOT**

To help children achieve the national guidelines for daily physical activity and screen time, OSHC providers are recommended to:

| **Session** | **Physical Activity** | **Recreational Screen Time*** |
| --- | --- | --- |
| **Before School Care** | Encourage children to engage in a variety of physical activities, of which **15 minutes or more** is energetic play. More is better. | No more than **30 minutes.** Less is better. |
| **After School Care** | Encourage children to engage in a variety of physical activities, of which **30 minutes or more** is energetic play. More is better. | No more than **60 minutes.** Less is better. |
| **Vacation Care** | Encourage children to engage in a variety of physical activities, of which **60 minutes or more** is energetic play spread throughout the day. More is better. | No more than **2 hours** of recreational screen time over the entire day. Less is better. |

*this does not include screen time to complete homework

**EXPANDED GUIDELINES FOR PHYSICAL ACTIVITY AND SCREEN TIME IN OSHC**

It is important that children are physically active every day to help them grow and develop. Whilst recreational screen time (e.g. TV, video games) is enjoyable, it is linked with poorer health, wellbeing and academic performance – as such, it should be limited.

Australian national guidelines recommend school-aged children **need at least 60 minutes** of moderative to vigorous physical activity every day. Additional physical activity, and particularly vigorous activity, is better. Children should get **no more than two hours per day** of recreational screen time. The activities that children do at OSHC play an important role in meeting daily physical activity and screen time guidelines.

Given that OSHC takes place during times of the day that would traditionally be children’s free time, opportunities for children to be active through varied, fun and play-based activities are encouraged. Children should be allowed to naturally move between varying levels of active play (ranging from light to energetic activities) and quiet pursuits. Quiet activities, such as reading, talking, arts and crafts and puzzles are good for children, and are preferable to recreational screen time.

**PHYSICAL ACTIVITY RECOMMENDATIONS**

The following strategies can help children achieve the recommended amount of physical activity at OSHC. It should be fun and provide opportunities for children of different ages and abilities.

- Free play usually involves a range of sedentary to light to energetic activities. For children to achieve the recommended amount of energetic play, more time for active play will be needed (e.g. to reach 30 minutes of energetic play, approx. 90 minutes of play time may be needed).
- Outdoor, free-play is an important source of children’s physical activity. OSHC services are encouraged to consider ways to offer outdoor play for as much of the care session as possible.
- Indoor spaces for energetic play are important during inclement (hot or wet) weather, and to increase the variety of activities available to children. OSHC services are encouraged to consider ways to offer indoor energetic play opportunities.
- Equipment and music can be offered to encourage active play (e.g. bats, balls, nature play construction materials and dancing music).
- Staff-led activities (e.g. playground games and sports) may be offered in addition to free play. Games that allow children to be active most of the time (e.g. Red Rover) are preferable to games that involve sitting out or extended waiting for a turn (e.g. tunnel ball).
- Children should be allowed to self-regulate and move freely between energetic play and more sedentary activities as they choose.
- Scheduling ample daily opportunities for active play and outdoor play may be a helpful strategy to achieve physical activity recommendations.

**SCREEN TIME RECOMMENDATIONS**

The following strategies can help children achieve the recommended amount of recreational screen time at OSHC. Children should be allowed to move freely between active play and quiet activities at OSHC. Quiet activities are important for children to relax, feel emotionally settled, and to learn.

- Children need engagement in activities other than recreational screen time; so it is important that an engaging program is offered to children in OSHC services to minimise boredom, unacceptable behaviour and a desire for screen time.
- Active play, and quiet non-screen activities (e.g. reading, talking, board games, arts and crafts) should be offered in preference to screen time.
- Many children, if offered recreational screen time concurrently with other types of activities, will choose screen time. Therefore, it is recommended that recreational screen time is only offered sparingly e.g. only offered on particular days (Fridays/last day of term) or during inclement weather.
- If recreational screen time is offered daily, the time should be restricted and given low priority compared with other activities (e.g. it could be made available during the last 30 minutes of the after school care session).
- Children should be discouraged from bringing electronic devices to OSHC, unless for homework purposes.
- Staff and older children should be encouraged to model appropriate screen time use (e.g. only access personal devices at beginning and end of sessions)

Note, using electronic devices to complete homework is not recreational screen time. The recommendations above apply to recreational screen activities such as watching TV and DVDs, and playing computer and electronic games.

**RECOMMENDATIONS FOR EDUCATORS**

OSHC Educators play a critical role in planning and delivering OSHC services, including setting and changing an OSHC service’s culture surrounding physical activity and screen time practices. It is important that educators understand the importance of physical activity and screen time to children’s health and wellbeing, and facilitate and model appropriate behaviours. The following educator focused strategies may help them to assist children in their care to achieve the recommended amount of physical activity and recreational screen time at OSHC.

- OSHC Educators have access to regular professional development which provides opportunities for them to develop their knowledge and skills to help children achieve healthy active behaviours e.g. through short online training modules
- It is recommended that the OSHC physical activity and screen time guidelines be embedded in orientation and annual reviews of all educators to ensure awareness and understanding of the guidelines
- It is recommended that the OSHC physical activity and screen time guidelines be embedded into OSHC services’ policies and procedures.
- When planning the OSHC program for the term/vacation period, attention should be given to prioritise physical activity over screen time every day, and efforts made to meet time specific recommendations every day.
- Educators should be encouraged to model healthy physical activity and screen time behaviours by engaging children during active play (e.g. actively supervising play activities and offering verbal encouragement) and minimising use of personal electronic devices.
- Ongoing training to develop OSHC educators’ skills to facilitate active games is encouraged.
