# Supplementary material for: Development of Australian physical activity and screen time guidelines for outside school hours care: an international Delphi study
Source: Int J Behav Nutr Phys Act. 2021 Jan 6;18:3. doi: 10.1186/s12966-020-01061-z (PMC7789289; doi:10.1186/s12966-020-01061-z)
Supplement: Supplementary file 3 — Additional file 3. [file 12966_2020_1061_MOESM3_ESM.docx]

**Table 4: CREDES Checklist Items***

| **Item** | **Manuscript Section** | **Page No.** |  |
| --- | --- | --- | --- |
| **Transparency and quality of reporting** |  |  |  |
| Purpose well defined | Background | 4 and 5 |  |
| Rationale for Delphi Selection of experts clearly justified | Methods | 6 |  |
| Clear description of methods | Methods | 7 - 10 |  |
| Flow chart Clear definition of consensus | Methods | 8 and figure 1 |  |
| Pilot test of instruments | Methods | 7 - 8 |  |
| Transparent reporting of results | Results | 11 – 15 and figure 1 |  |
| Data analysis clearly justified and reported Information of rounds | Methods | 11 |  |
| Discussion of limitations | Discussion | 20 |  |
| Adequacy of conclusions | Conclusion | 22 |  |
| **Selection criteria expert panel** | Methods | 6 |  |
| Member of organisation |  |  | 🗸 |
| Recognised authority |  |  | 🗸 |
| Relevant clinical academic expertise |  |  | 🗸 |
| Geographical scope |  |  | 🗸 |
| Setting/work field |  |  | 🗸 |
| Profession/ stakeholder |  |  | 🗸 |
| **Definition of Consensus** | Likert scale (1-9). Consensus was defined a priori as ≥ 80% of respondents rating an item as “critically important” ( score 7 – 9). | | |

*****Taken from Table 5 – Selection of experts, definitions of consensus and quality of reporting - Jünger S, Payne SA, Brine J, Radbruch L, Brearley SG. Guidance on Conducting and REporting DElphi Studies (CREDES) in palliative care: Recommendations based on a methodological systematic review. Palliative Medicine. 2017;31(8):684-706. PubMed PMID: 28190381.
